# BA-ATEMNet: Bayesian Learning and Multi-Head Self-Attention for Theoretical Denoising of Airborne Transient Electromagnetic Signals

**DOI:** 10.3390/s25010077

**Published:** 2024-12-26

**Authors:** Weijie Wang, Xuben Wang, Xiaodong Yu, Debiao Luo, Xinyue Liu, Kai Yang, Wen Yang, Xiaolan Yang, Ke Hu, Wenyi Hu

**Affiliations:** 1 School of Geophysics, Chengdu University of Technology, Chengdu 610059, China; wangweijie@cdu.edu.cn (W.W.); yangkai@cdu.edu.cn (K.Y.); 2 Information Network Center, Chengdu University, Chengdu 610106, China; luodb@cdu.edu.cn (D.L.); liuxy@cdu.edu.cn (X.L.); yangwen@cdu.edu.cn (W.Y.); yangxiaolan@cdu.edu.cn (X.Y.); huke@cdu.edu.cn (K.H.); huwenyi@cdu.edu.cn (W.H.); 3 School of Computer Science, Chengdu University, Chengdu 610106, China; yuxiaodong@cdu.edu.cn; 4 Key Laboratory of Digital Innovation of Tianfu Culture, Sichuan Provincial Department of Culture and Tourism, Chengdu University, Chengdu 610106, China

**Keywords:** deep learning, airborne transient electromagnetic method, multi-head self-attention mechanism, Bayesian learning, signal denoising

## Abstract

Airborne transient electromagnetic (ATEM) surveys provide a fast, flexible approach for identifying conductive metal deposits across a variety of intricate terrains. Nonetheless, the secondary electromagnetic response signals captured by ATEM systems frequently suffer from numerous noise interferences, which impede effective data processing and interpretation. Traditional denoising methods often fall short in addressing these complex noise backgrounds, leading to less-than-optimal signal extraction. To tackle this issue, a deep learning-based denoising network, called BA-ATEMNet, is introduced, using Bayesian learning alongside a multi-head self-attention mechanism to effectively denoise ATEM signals. The incorporation of a multi-head self-attention mechanism significantly enhances the feature extraction capabilities of the convolutional neural network, allowing for improved differentiation between signal and noise. Moreover, the combination of Bayesian learning with a weighted integration of prior knowledge and SNR enhances the model’s performance across varying noise levels, thereby increasing its adaptability to complex noise environments. Our experimental findings indicate that BA-ATEMNet surpasses other denoising models in both single and multiple noise conditions, achieving an average signal-to-noise ratio of 37.21 dB in multiple noise scenarios. This notable enhancement in SNR, compared to the next best model, which achieves an average SNR of 36.10 dB, holds substantial implications for ATEM-based mineral exploration and geological surveys.

## 1. Introduction

The Airborne Transient Electromagnetic Method (ATEM) utilises a flying platform as a means of transporting the requisite apparatus for the collection of the electromagnetic response signal from the designated exploration area. This method permits the undertaking of underground geological exploration without the need for ground personnel to approach the designated area of investigation. The method is applicable in regions characterized by intricate topography, including elevated terrain, arid zones, wetlands, and forested areas. ATEM technology is employed extensively in a number of fields, including mineral resources, hydrology, geological mapping, and environmental protection [1]. However, one of the most significant challenges facing this technology is the effective denoising and interpretation of the collected weak secondary field signals. The noise sources are diverse and complex in nature, including Gaussian noise from the surrounding environment, errors introduced by instrumentation, and atmospheric noise resulting from electromagnetic radiation in the atmosphere [2].

In the past, traditional noise reduction techniques, such as Wiener filtering and wavelet transformation, were effective in simple and static noise environments. However, in complex and dynamic noise environments, the results of these methods are frequently inadequate [3]. In order to address this challenge, researchers have developed a series of statistical methods designed to address these issues. Such techniques include Principal Components Analysis (PCA) [4] and Minimum Noise Fraction (MNF) [5]. Principal Component Analysis (PCA) is a statistical technique that can be employed to reduce the impact of noise by means of dimensionality reduction. Meanwhile, MNF enables the system to more effectively separate signal and noise by transforming the data into a new coordinate system and sorting it according to the contribution of noise.

The advent of computing technology has given rise to the development of novel denoising techniques, including Kernel Minimum Noise Fraction (KMNF) [6] and Noise-Whitening Weighted Nuclear Norm Minimisation (NW-WNNM) [7]. KMNF employs kernel methods to enhance the effectiveness of MNF in addressing nonlinear data relationships. In contrast, NW-WNNM optimises the global nuclear norm through the optimisation of the global nuclear norm minimisation problem, effectively removing noise while preserving the complexity of the geological structure.

The field of neural networks is undergoing a period of rapid development in the area of image denoising methods. Zhang et al. proposed a deep convolutional neural network [8] (Denoising Convolutional Neural Networks, DnCNN) that markedly accelerates training and enhances denoising effects by integrating residual learning and batch normalisation techniques. Subsequently, Zhang et al. proceeded to optimise the network further, introducing the Fast and Flexible Denoising Convolutional Neural Network (FFDNet) [9]. The FFDNet network incorporates an adjustable noise level map, which enables the network to process images with varying noise levels in an efficient and effective manner, particularly in contexts where complex noise is present in practical applications.

The Transformer structure, as proposed by Vaswani et al., has had a profound impact on the field of information processing, as it relies entirely on self-attention mechanisms to handle sequence tasks [10]. The model’s multi-head attention mechanism enables the simultaneous focus on information from disparate representation subspaces, thereby markedly enhancing processing efficiency and training accuracy. In a recent study, Tian et al. proposed the Cross Transformer Denoising Convolutional Neural Network (CTNet) [11], which employs the Transformer mechanism to interact with a multilayer network, thereby enabling the extraction of structural information and the identification of relevant data between pixels. This approach enhances the model’s capacity to adapt to complex scenes and improves its denoising efficiency. Moreover, some researchers have conducted comprehensive research on attention mechanisms and applied them to image denoising. The Adaptive Dual Self-Attention Network (IDEA-Net) [12] model, proposed by Zuo et al., markedly enhances the efficacy of single-image denoising through the integration of an adaptive dual self-attention mechanism.

Xue et al. employed dictionary learning to denoise airborne electromagnetic data noise [13]. Xue et al. employed a dictionary learning approach to denoise airborne EM data, utilising a neural network to learn and adapt to the EM data dictionary. This approach effectively separates the signal from the noise through the utilisation of sparse representation techniques [14]. Wu et al. developed a deep learning method based on a denoising auto-encoder (DAE), which enables the DAE to accurately distinguish between signal and noise by combining simulated responses and actual noise data training sets. This demonstrates the potential of deep learning in the processing of electromagnetic data [15]. Sun et al. developed a preprocessing method to suppress power harmonic noise in urban drag transient electromagnetic data, utilising an improved genetic algorithm to facilitate a rapid search [16]. Wu et al. employed an autoencoder denoising method based on a long short-term memory network (LSTM) to effectively remove multi-source noise by modeling long-term dependencies [17].

The TEM-NLnet method, developed by Wang et al., employs a Generative Adversarial Network (GAN) to learn the noise characteristics present in real environments. This approach permits the construction of noise–signal pairing datasets for deep network training, which maintains a high denoising performance under different noise distributions and levels [18].

Sun et al. combined the MNF algorithm and a deep neural network. Following the application of MNF to enhance the signal-to-noise ratio, the spatial and temporal characteristics of the signal were extracted by the neural network, resulting in the effective suppression of random noise [19]. The Convolutional Neural Network and Gated Recurrent Unit Dual Autoencoder (CNN-GRU Dual Autoencoder, CG-DAE) method, proposed by Yu et al., combines a convolutional neural network with a gated recurrent unit. This results in enhanced data processing efficiency and a more effective denoising effect through the dual autoencoder structure [20].

However, the TEM is a prevalent technique employed in mineral exploration and geological surveys, and interpreting TEM data can be quite challenging due to the interference and noise present. Conventional approaches for denoising TEM data, like wavelet denoising and total variation denoising, have proven effective in mitigating noise; however, they possess certain limitations. Wavelet denoising is predicated on the assumption that the noise is Gaussian and stationary, which does not always hold true for actual TEM data. Conversely, total variation denoising can be computationally intensive and may not properly conduct noise that lacks sparsity.

Recently, deep learning techniques have emerged for denoising TEM data, including CNNs and RNNs. However, these approaches also exhibit limitations. CNNs struggle to capture long-range dependencies within the data, while RNNs can be resource-intensive and may face problems with vanishing gradients.

To resolve the mentioned problems, a novel method is proposed for denoising TEM data that provides a Bayesian hybrid framework with a multi-head attention mechanism. The Bayesian approach facilitates the modeling of uncertainty associated with both noise and signal, while the multi-head attention mechanism allows for the effective capture of long-range dependencies in the data. The suggested method surpasses the efficiency of existing techniques during a real-world TEM dataset. The introduction critically examines the shortcomings of both traditional and deep learning-based methods, presenting the suggested solution as a means to resolve these issues.

In view of the limitations of existing methodologies and the potential of emerging techniques, this study puts forward the BA-ATEMNet model, which combines Bayesian learning and multi-head self-attention mechanisms. This synergy enables the model to demonstrate enhanced denoising precision in complex noise environments, and to adapt to unknown noise effectively. Moreover, the parallel processing capability of multi-head self-attention in the model can significantly improve data processing speed and accuracy.

The following sections will provide a comprehensive description of the architectural design of the BA-ATEMNet model, an in-depth analysis of its core technical aspects, and a detailed account of its deployment in diverse noisy environments. The principal contributions of this study can be summarised as follows:

- A deep learning model that incorporates a multi-head self-attention mechanism. This study presents the construction of a deep learning model that integrates a multi-head self-attention mechanism with the objective of enhancing the feature extraction capacity of convolutional neural networks in the context of noise level identification. The parallel processing of multiple attention heads enables the model to more accurately distinguish between noise and signal, thereby improving the accuracy of noise level estimation and denoising performance. This technique is particularly well-suited to the processing of large-scale electromagnetic datasets with complex noise backgrounds, thereby enhancing the accuracy of data cleaning.
- A structured model is designed with the objective of incorporating Bayesian priors. This study employs a model design based on Bayesian priors to construct a prediction framework with enhanced uncertainty management capabilities. This approach not only enhances the model’s interpretability, but also optimises the learning and analysis of signals in complex electromagnetic environments through the incorporation of prior knowledge.
- A general denoising strategy for use in a multi-noise environment is as follows: This study proposes a denoising framework for a composite noise environment comprising Gaussian white noise and sky noise with randomly varying noise levels. In contrast to conventional denoising techniques, which are designed to address a single noise source, this model demonstrates its processing capabilities when multiple noise sources coexist. This method improves denoising performance and is particularly effective for removing multiple noise interferences, which are commonly encountered in real-world environments. Consequently, the model is highly adaptable and robust.

## 2. Preliminary

### 2.1. Theory of Airborne Transient Electromagnetic

ATEM is a technique that is widely employed in the field of geophysical exploration. The technique relies on sophisticated electromagnetic theories and numerical simulation methods to detect and map underground structures. In ATEM, devices carried by an aircraft or helicopter emit electromagnetic pulses that penetrate the Earth’s surface and excite induced currents in underground structures. The resulting electromagnetic fields then generate further fields, the characteristics of which decay in a manner closely related to the electrical conductivity of the underground materials. The detection of the decay process of the induced electromagnetic fields with receivers carried on an airborne platform allows the inference of information about the underground structure [21].

Maxwell’s equations represent the foundational theoretical framework that describes electromagnetic fields and waves. In addition, they serve as the foundation for the development of the ATEM forward and inverse models [22]. The fundamental Maxwell equations are as follows:

(1) $$\begin{array}{c} \nabla \times e+\frac{𝜕b}{𝜕t}=0\\ \nabla \times h-\frac{𝜕d}{𝜕t}=j\\ \nabla \cdot b=0\\ \nabla \cdot d=\rho \end{array}$$

where ρ (C/m^3^) is the charge density; vector b (Wb/m^2^) is the magnetic flux density, i.e., the magnetic induction intensity; vector **h** (A/m) is the magnetic field intensity; vector **d** (C/m^2^) is the electric flux density, i.e., the potential shift; vector **e** (V/m) is the electromagnetic intensity; **j** (A/m^2^) denotes the current density; and ∂/∂t denotes the rate of change with time.

When applying the Maxwell’s system of equations to geophysical problems, it is necessary to consider its correlation with electromagnetic properties such as electrical conductivity(σ), magnetic permeability(μ), and dielectric constant(ε) of the subsurface medium. The set of Maxwell’s equations of Equation (1) can be related through the intrinsic relationship between them:

(2) $$\begin{array}{c} d=\varepsilon e\\ b=\mu h\\ j=\sigma e \end{array}$$

In a one-dimensional forward model, it is assumed that the strata are horizontally distributed, that each layer has a uniform conductivity, that electromagnetic waves propagate vertically in the model, and that the electromagnetic properties of each layer can be calculated separately. The transmitter utilises a central loop source. As outlined in Yu’s paper, following the simplification of the homogeneous layered medium, the assumption of quasi-static conditions, the resolution of the boundary value problem, and other necessary steps, the formula for the vertical magnetic field component of the central loop source can be derived as follows:

(3) $$H_{z}=\frac{Ia}{2}\int_{0}^{\infty }\left[e^{-\mu_{0}\left(z+h\right)}+r_{TE}e^{\mu_{0}\left(z-h\right)}\right]\frac{\lambda^{2}}{\mu_{0}}J_{1}\left(\lambda a\right)d\lambda$$

where *I* is the emission current, *a* is the radius of the emission coil, λ is the integration variable, and $r_{TE}$ is the reflection coefficient.

(4) $$r_{TE}=\frac{\lambda -{\hat{\mu }}_{1}}{\lambda +{\hat{\mu }}_{1}}$$

${\hat{\mu }}_{1}$ is obtained from a recursive formula. In the case of transceivers with receiving and transmitting lines that are in substantial overlap, the actual observed electromagnetic response value should be the induced electromotive force in the receiving coil. In his paper, Yu derives the induced electromotive force in the vertical direction of the receiving coil in the frequency domain, as follows [23]:

(5) $$V_{z}=-\frac{i\omega \mu_{0}S_{Rx}Ia}{2}\int_{0}^{\infty }\left[e^{-\lambda \left(z+h\right)}+r_{TE}e^{\lambda \left(z-h\right)}\right]\lambda J_{1}\left(\lambda a\right)d\lambda$$

In general, deriving an analytical solution for induced electromotive force represents a significant challenge. Consequently, numerical calculation methods are typically employed for such calculations. The direct calculation of the electromagnetic response from the time domain is a complex and computationally intensive process for time-domain electromagnetic methods. Accordingly, the electromagnetic response in the frequency domain is typically calculated initially, and subsequently, the frequency–time conversion method is employed to transform the electromagnetic response in the frequency domain into the corresponding electromagnetic response value in the time domain.

### 2.2. Bayesian-Based Signal-to-Noise Ratio Weighted Prior Theory

Suppose there is a noisy image *y*, and the noise n is additive Gaussian noise $n∼N\left(0,\sigma_{n}^{2}\right)$. Then, *y* can be expressed as the sum of the clean image *x* and the noise *n*, as follows:

(6) y=x+n

Assume that the clean image x also obeys a Gaussian distribution $x∼N\left(\bar{x},\sigma_{x}^{2}\right)$ where $\bar{x}$ is the mean and $\sigma_{x}^{2}$ is the variance. From this, we can see that

(7) $$y|x∼N\left(x,\sigma_{n}^{2}\right)$$

According to the Bayesian formula [24]:

(8) $$p\left(x|y\right)=\frac{p\left(y|x\right)\cdot p\left(x\right)}{p\left(y\right)}$$

where $p\left(x|y\right)$ is the probability that given the data y, the probability that the hypothesis *x* holds given the data is called the posterior probability; $p\left(y|x\right)$ is the probability that the re-assumption x holds, the probability of the observed data *y*, called the likelihood probability; $p\left(x\right)$ is the a priori probability that the assumption *x* holds. We expect to find the maximum a posteriori probability estimate ${\hat{x}}_{MAP}=\arg\underset{x}{\max}p\left(x|y\right)$ to predict a clean image. Since *x* and y∣x both obey Gaussian distributions, it follows that:

(9) $$p\left(x\right)=\frac{1}{\sqrt{2\pi \sigma_{x}^{2}}}e^{-\frac{{\left(x-\bar{x}\right)}^{2}}{2\sigma_{x}^{2}}}$$

(10) $$p\left(y|x\right)=\frac{1}{\sqrt{2\pi \sigma_{n}^{2}}}e^{-\frac{{\left(y-x\right)}^{2}}{2\sigma_{n}^{2}}}$$

Since the product of two independent Gaussian distributions is still a Gaussian function, the probability distribution of the posterior is also Gaussian, and is obtained by multiplying Equations (9) and (10):

(11) $$p\left(x|y\right)\propto e^{-\frac{{\left(y-x\right)}^{2}}{2\sigma_{n}^{2}}}\cdot e^{-\frac{{\left(x-\bar{x}\right)}^{2}}{2\sigma_{x}^{2}}}$$

We expand and rearrange Equation (11) to obtain:

(12) $$p\left(x|y\right)\propto e^{-\left(\frac{1}{2\sigma_{n}^{2}}{\left(y-x\right)}^{2}+\frac{1}{2\sigma_{x}^{2}}{\left(x-\bar{x}\right)}^{2}\right)}$$

Organizing Equation (12) into quadratic form for *x* and completing the method of collocation of the squared terms [25], we obtain:

(13) $$p\left(x|y\right)\propto e^{-\left(\frac{\sigma_{x}^{2}+\sigma_{n}^{2}}{2\sigma_{x}^{2}\sigma_{n}^{2}}x^{2}-\frac{2\sigma_{n}^{2}\bar{x}+2\sigma_{x}^{2}y}{2\sigma_{x}^{2}\sigma_{n}^{2}}x\right)}$$

In order to find the maximum a posteriori estimate of *x*, it is necessary to find the optimal value of *x* by taking the derivative of the maximisation Formula (13) and making the derivative zero:

(14) $$\hat{x}=\frac{\sigma_{n}^{2}\bar{x}+\sigma_{x}^{2}y}{\sigma_{x}^{2}+\sigma_{n}^{2}}$$

It is also known that the signal-to-noise ratio formula $S=\frac{\sigma_{x}^{2}}{\sigma_{n}^{2}}$, so $\hat{x}$ is expressed by the signal-to-noise ratio formula results, as follows:

(15) $$\hat{x}=\bar{x}\frac{1}{1+S}+y\frac{S}{1+S}$$

Equation (15) illustrates that a dynamic balance can be attained in image denoising through the adjustment of a weighted signal-to-noise ratio. In the case of a low noise level, that is to say, a high value of S, the contribution of the observed data y to the final estimate is increased; conversely, when the noise level is high, greater reliance is placed on prior knowledge. This method offers an effective balancing strategy for image recovery in the presence of diverse noise conditions.

### 2.3. Multi-Head Self-Attention Theory

As outlined in Ref. [26], self-attention represents a specific type of attention mechanism that enables each moment model to perceive global information in a dynamic manner and direct its focus towards the most pertinent information at any given moment. Self-attention links disparate positions within a single sequence, thus enabling the model to process each element of the sequence in the context of all other elements within the sequence.

First, calculate the query (Query), key (Key), and value (Value) with the following formula:

(16) $$Q=XW^{Q},K=XW^{K},V=XW^{V}$$

where X is the input sequence, Q is the query matrix, K is the key matrix, and V is the value matrix. $W^{Q},\;W^{K},\;W^{V}$ are the learnable weight matrices of Q, K, and V, respectively.

We use the attention function to compute the dot product of the query with all the keys and divide by $\sqrt{d_{k}}$ and apply the softmax function to get the weights on the values with the following formula:

(17) $$\mathrm{Attention}\left(Q,K,V\right)=\mathrm{soft}\max\left(\frac{{\mathrm{QK}}^{T}}{\sqrt{d_{k}}}\right)V$$

where $d_{k}$ is the dimension of the key and is used to scale the result of the dot product to prevent gradients from vanishing or exploding.

The multi-head attention mechanism extends the basic attention mechanism to enable the model to focus on information in parallel in different subspaces of representation [27]. The aforementioned process can be elucidated through the following steps and equations:

Step 1: Calculate attention for each head separately.

For each head i, we compute it separately:

(18) $$Q_{i}={\mathrm{QW}}_{i}^{Q},K_{i}={\mathrm{KW}}_{i}^{K},V_{i}={\mathrm{VW}}_{i}^{V}$$

Step 2: Scaled dot-product attention for each head.

For each head i, the basic attention formula is implemented:

(19) $$\mathrm{head}=\mathrm{Attention}\left({\mathrm{QW}}_{i}^{Q},{\mathrm{KW}}_{i}^{K},{\mathrm{VW}}_{i}^{V}\right)$$

Step 3: Merge the output of all heads.

The outputs of all the heads are then spliced together and passed through a final linear layer:

(20) $$\mathrm{MultiHead}\left(Q,K,V\right)=\mathrm{Concat}\left({\mathrm{head}}_{1},\cdots ,{\mathrm{head}}_{h}\right)W^{o}$$

where $W^{O}$ is another learnable weight matrix.

The multi-head attention mechanism enables the model to capture a diverse range of features present within the sequence, operating across a number of distinct representation subspaces. Each head is capable of focusing on a specific aspect of the sequence, thereby facilitating a more comprehensive understanding of the input data.

## 3. The Proposed Method

### 3.1. Network Architecture

**Figure 1:**
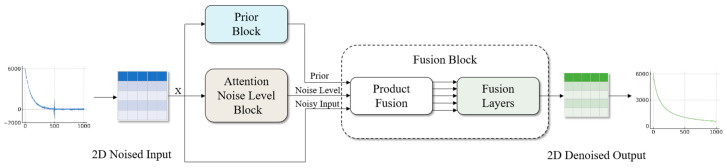
Overall structure diagram of BA-ATEMNet model.

This paper puts forth a deep learning network model based on Bayesian learning with multi-head self-attention, designated as BA-ATEMNet, as a means of more effectively eliminating noise in aviation transient electromagnetic signals. The model fuses the TEMDNet [28] and Blind Universal Image Fusion Denoiser frameworks, enhancing the denoising capability through the introduction of Bayesian learning and attention mechanisms. The overall structure of the BA-ATEMNet model is illustrated in Figure 1. Once the aviation transient electromagnetic signals have been loaded into the memory map and normalised, the one-dimensional electromagnetic signals are converted into a 32 × 32 two-dimensional matrix by the reshaping method, which is then used as the input of BA-ATEMNet. Subsequently, the two-dimensional matrix output from the network structure is converted back to a one-dimensional airborne transient electromagnetic signal by the inverse shaping method, thereby completing the denoising task of electromagnetic signals. The BA-ATEMNet network structure comprises three distinct parts: the Prior Block, the Attention Noise Level Block, and the Fusion Block.

- The Prior Block is employed for the capture of multi-scale features, utilising dilated convolutional layers and residual blocks to provide high-quality prior information for the denoising process.
- The Attention Noise Level Block is employed to extract preliminary features through a convolutional layer, enhance the feature representation using a multi-head self-attention mechanism, and facilitate more accurate capture of complex noise patterns in the data.
- The Fusion Block is employed for the integration of the outputs of the Prior Module and the Attentional Noise Level Module. The final denoising effect is optimised through the implementation of prior integration with signal-to-noise ratio weighting, which is achieved through product fusion and a fusion layer.

### 3.2. The Prior Block

**Figure 2:**
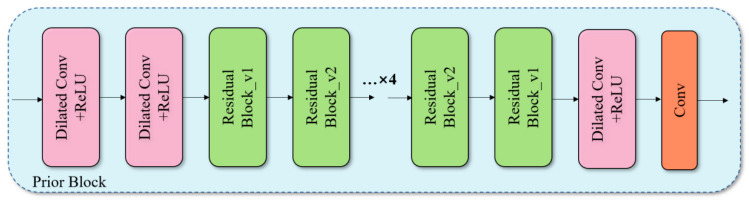
Structure diagram of Prior Block.

The design of the Prior Block is intended to facilitate the extraction and enhancement of useful signal features in deep denoising networks, while simultaneously suppressing unnecessary noise components. The structure of this block is illustrated in Figure 2 and comprises the following elements:

- The configuration of the Dilated Conv+ReLU block is as follows: The aforementioned block is located at the initial stage of the Prior Block. This approach permits an expansion of the receptive field by dilating the kernel step size without an accompanying increase in the computational burden. This results in the capture of a more extensive range of contextual information, which in turn leads to a considerable enhancement in the accuracy of signal feature extraction. The positioning of this block at the final stage of the Prior Block enables additional processing and refinement of the output features, optimisation of the quality of the feature map, and the provision of clear features for denoising. The rectified linear unit (ReLU) function introduces a nonlinearity to the network, thereby enhancing its expressive power.
- The Residual Block is defined as follows: The incorporation of skip connections addresses the issue of gradient disappearance in deep networks, thereby ensuring the efficacy and stability of the network structure. Each Residual Block comprises two convolutional layers with ReLU activation and batch normalisation, which facilitate accelerated training and enhanced generalisation ability. Moreover, the repetition of the Residual Block enables the extraction of complex features within the electromagnetic signal at varying depths. This process may enhance the feature response of a specific frequency, rendering it particularly well-suited for the processing of electromagnetic signals exhibiting multi-scale changes.

### 3.3. The Attention Noise Level Block

**Figure 3:**
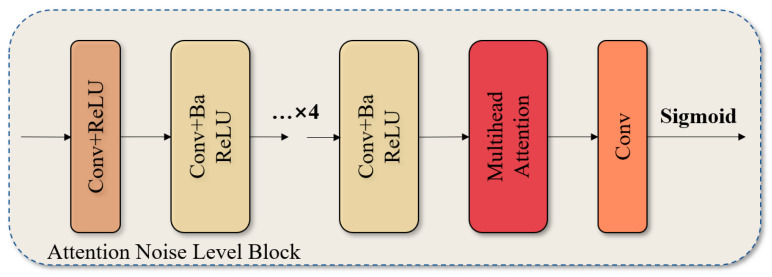
Structure diagram of the Attention Noise Level Block.

The Attention Noise Level Block represents a significant advancement in the model. The model employs a multi-head self-attention mechanism to ensure the precise regulation of feature weights and optimise the separation efficiency of noise and signal. The module structure is depicted in Figure 3 and comprises the following elements:

- Conv+ReLU: This block begins with the use of a multi-layer convolutional network, with each layer followed by a ReLU activation function to extract the underlying features of the input signal. These layers not only enhance the model’s initial understanding of the raw input data, but also help capture more complex data patterns through nonlinear transformations, laying the foundation for advanced feature processing.
- The Conv+Ba ReLU block is defined as follows: The block applies batch normalisation after each convolutional layer, thereby accelerating the convergence of model training and enhancing the model’s generalisation ability with respect to diverse datasets. The implementation of batch normalisation on feature maps has the effect of reducing internal covariate shifts and enhancing the stability of the network.
- The concept of Multi-head Attention can be defined as follows: This constitutes the fundamental innovation of the block, which enables the model to process information in disparate representation subspaces in parallel, and to re-weight the feature map by calculating the dependencies between different features. Each “head” focuses on a distinct combination of features, and this design allows the network to capture the nuances between noise and signal in a more comprehensive manner, while maintaining a low computational complexity.
- The activation function employed for the sigmoid is as follows: Subsequent to multi-head attention, the final convolutional layer employs a sigmoid activation function, which enables the normalisation of the output to the [0, 1] interval and furnishes suitable feature weights for the subsequent stage of noise suppression and signal reconstruction.

This structure markedly enhances the model’s capability to adapt to a heterogeneous array of noise conditions by accurately regulating and optimising the assignment of weights to features. In practice, this enables the model to effectively identify and extract noise levels of varying types and intensities, while maintaining the signal details in their original form.

### 3.4. The Fusion Block

**Figure 4:**
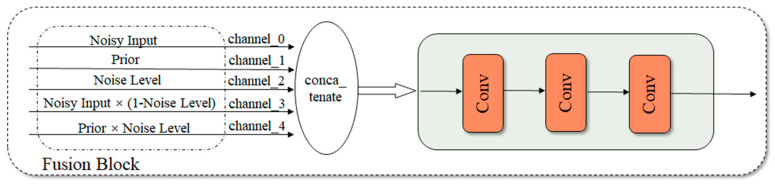
Structure diagram of the Fusion Module.

The Fusion Block represents a pivotal component of the BA-ATEMNet model. Its primary function is to facilitate the intelligent fusion of information derived from both the a priori module and the attentional noise level module. This enables the achievement of efficient signal-to-noise weighted denoising. Prior to the a priori knowledge being fused into the final denoising result, the corresponding weights are adjusted and optimised in accordance with the environmental noise level. This further enhances the denoising performance of the model. The structure and working mechanism of the block are illustrated in Figure 4. The specific operation of the Fusion Block is as follows:

Multi-Channel Input: Input from multiple channels. The Fusion Block is capable of receiving five distinct inputs, each of which corresponds to a different signal processing channel.

▪ Channel 0: This channel inputs raw noise without any pre-processing (Noisy Input).
▪ Channel 1: This channel inputs signals (Prior) that have been processed by the Prior Block.
▪ Channel 2: This channel inputs the Noise Level, which reflects the estimated level of noise in the input signal.
▪ Channel 3: This channel inputs the product of Noisy Input and the Noise Level (1-Noise Level). This input helps reduce the effect of noise in the signal.
▪ Channel 4: This channel inputs the product of Prior and Noise Level. This input content helps to strengthen the signal component in the noise characteristics.
• Feature fusion (conca_tenate): The signals from all channels are fused by combining the feature vectors of each channel into a unified one-dimensional feature space using a concatenation method. This ensures that information from different processing stages can be fully utilised in subsequent processing steps.
• Multiple convolutional layers: The features after feature fusion are processed through a number of convolutional layers. The task of these convolutional layers is to integrate the input features, extract the information most beneficial for denoising, and suppress residual noise. This step refines and clarifies the final output by adjusting and optimising the relationship between features.
• Output: after successive convolutional processing, the Fusion Block outputs the final denoised signal.

The design of the aforementioned modules allows our model to utilise a priori information and an attention mechanism in order to effectively remove noise from electromagnetic response data. The organic combination between each module enables the model to maintain good denoising performance, even when dealing with complex noise environments.

## 4. Experiments

As aforementioned, the integration of Bayesian theory with the multi-head attention mechanism within a network entails several key steps. Bayesian theory serves as a statistical framework that facilitates the mathematical updating of the probability of a hypothesis in light of new data. In deep learning, it is instrumental in modeling the uncertainty associated with a neural network’s predictions. Conversely, the multi-head attention mechanism is designed to enable a model to simultaneously focus on information from various representation subspaces across different positions. This is achieved by linearly projecting the queries, keys, and values, followed by the computation of attention weights. To merge these two methodologies, we begin by establishing a Bayesian neural network (BNN) that captures the uncertainty inherent in the predictions of the neural network. This network is characterized by its probabilistic nature, producing a probability distribution over potential output.

We proceed to establish a multi-head attention mechanism that enables the model to simultaneously focus on information from various representation subspaces across different positions. Subsequently, the Bayesian neural network has been integrated with the multi-head attention mechanism by using the attention weights to derive Bayesian attention, a probabilistic attention mechanism that accounts for the uncertainty inherent in the attention weights. Ultimately, the Bayesian attention has been employed to calculate the Bayesian multi-head attention, which serves as a probabilistic attention mechanism that captures the uncertainty of the attention weights for each individual head. The architecture of the network comprises an input sequence of tokens, each represented as a vector, which is processed through the Bayesian neural network, followed by the multi-head attention mechanism, Bayesian attention, and Bayesian multi-head attention, culminating in the output of the network, which is the Bayesian multi-head attention.

### 4.1. Datasets

(1) One-dimensional orthogonal electromagnetic response data generation.

In order to generate accurate one-dimensional forward electromagnetic response data, this study employed the GA-AEM model developed by Geoscience Australia. This model has been developed with the specific purpose of processing airborne electromagnetic data. It is capable of simulating and analysing electromagnetic responses, thereby supporting the interpretation of complex geological structures. All simulations were conducted using the GA-AEM model, the source code for which is publicly available on GitHub [29]. In this study, approximately 700,000 data points were generated with the objective of simulating and reflecting the electromagnetic properties in different geological environments in detail. The following section delineates the particular settings for the model parameters.

The resistivity range is as follows: the resistivity range extends from 5 to 500 Ω·m, encompassing a diverse array of geological environments, from highly conductive to low conductivity.

- The geological structure comprises multiple layers. The system allows for the creation of up to four geological layers, which can be used to simulate complex underground structures.
- The depth of the aforementioned geological structure is as follows: the maximum depth of each layer is 200 metres, with the thickness of each layer varying from 5 metres to 100 metres.
- The height of the inductor is as follows: the height is set between 40 and 60 metres in order to simulate the effect of different flight altitudes on the electromagnetic response.

Moreover, a standardised model structure comprising 25 fixed layers of depth was constructed, with the conductivity value of each layer converted into a 25-dimensional vector by interpolation. During the forward simulation, the electromagnetic response data were collected at 0.06-ms intervals between 0.06 and 6.64 ms after the emitted current ceased, resulting in a total of 24 data points. Subsequently, the data points were expanded to 1024 points through cubic spline interpolation for the purpose of training the deep learning model.

(2) Noise simulation.

**Figure 5:**
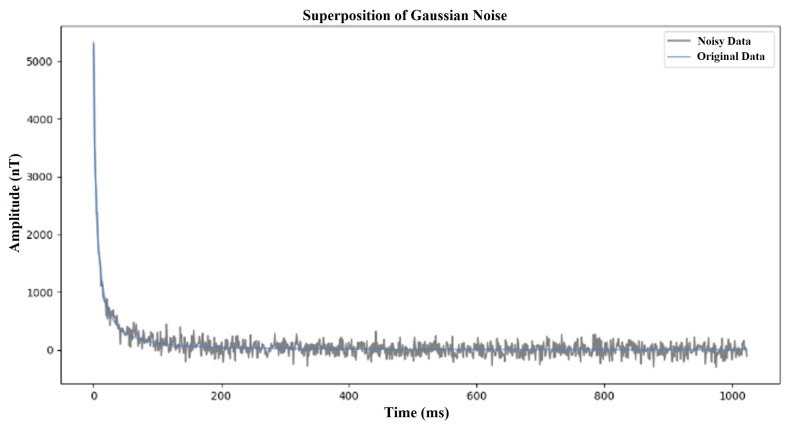
Simulation diagram of Gaussian noise.

**Figure 6:**
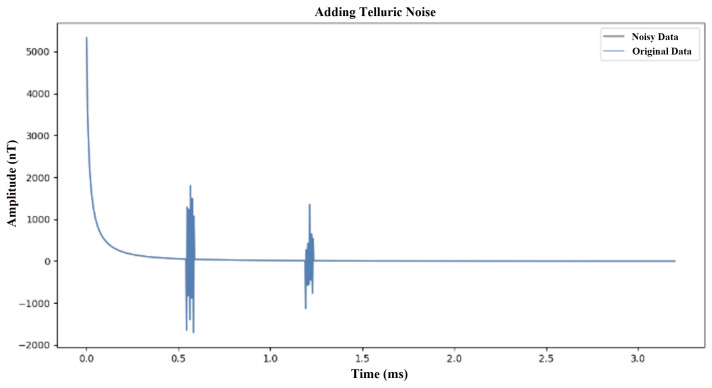
Simulation diagram of atmospheric noise.

In order to make the model effectively deal with noise interference in practical applications, we have introduced two main types of noise into the generated electromagnetic response data:

- Gaussian noise: This noise simulates the random fluctuations caused by instrument errors, with zero mean and a standard deviation of 50 to 200. This noise is added to the clean signal at random to reflect the instrument noise that may be encountered in actual observations. The Gaussian noise simulation is shown in Figure 5.
- Atmospheric noise: This noise simulates short-term transient noise caused by space weather activities. The uncertainty and irregularity of this noise make it necessary for the model to have stronger anti-interference ability and adaptability when dealing with actual situations. Therefore, in model development and testing, the introduction of space weather noise as an evaluation index under extreme conditions can help improve the performance and reliability of the model in complex environments.The Atmospheric noise simulation is shown in Figure 6.

By incorporating Gaussian noise and atmospheric noise into the training samples, the network is conditioned to enhance its robustness and adaptability to various noise types. This methodology, referred to as “data augmentation,” is a prevalent strategy in deep learning aimed at enhancing a model’s generalisability. Through exposure to diverse noise types during the training phase, the network acquires the ability to identify and eliminate noise patterns that are not inherent to the training dataset.

Consequently, the network is more likely to excel when confronted with new, unseen data that may exhibit different noise characteristics. In this instance, the BA-ATEMNet model is trained on a dataset that encompasses both Gaussian noise and atmospheric noise, which are prevalent forms of interference that can impact ATEM signals.

By training with this dataset, the model becomes adept at recognising and mitigating these specific noise types, thereby increasing its likelihood of performing effectively on new data that presents similar noise challenges. Nevertheless, it is important to acknowledge that the model’s efficacy with signals affected by other complex environmental noise may fluctuate. The model’s capacity to generalise to novel noise types is contingent upon the resemblance between the noise patterns present in the training data and those found in the new data.

In general, a more varied training dataset correlates with a greater likelihood of the model being resilient to different noise types. Therefore, if the objective is to utilise the model for signals influenced by other complex environmental noise, it would be advantageous to incorporate a broader spectrum of noise patterns into the training dataset. Several potential strategies to further enhance the model’s resilience to various noise types include:

- Expanding the training dataset to include additional noise types, such as electromagnetic interference (EMI) and radio-frequency interference (RFI).
- Implementing advanced data augmentation strategies, including noise injection and signal corruption.
- Employing transfer learning or domain adaptation methods to tailor the model for new noise types.
- Utilising more robust loss functions or regularization techniques to enhance the model’s generalizability.

In summary, while the BA-ATEMNet model demonstrates a talented capacity to generalise to signals affected by diverse complex environmental noise, further evaluation and testing are essential to assess its performance in practical applications.

(3) Dataset partitioning.

The generated dataset is divided into a training set, a validation set, and a test set, representing 70%, 15%, and 15% of the total data, respectively. This division strategy aims to ensure the generalisation ability and stability of the model on different data, while also allowing the actual denoising effect of the model to be evaluated on an independent test set.

### 4.2. Implementation Details

In this study, the Root Mean Square Error (RMSE) was used as the loss function for model training to accurately measure the deviation between the predicted output and the true target. The optimisation algorithm chosen was Adam, an adaptive estimation-based method that adjusts the learning rate of each parameter, which is particularly effective for handling large datasets with sparse gradients.

Learning rate adjustment: The initial learning rate was set at 0.001 and dynamically adjusted using the ReduceLROnPlateau scheduler. This scheduler monitors the validation loss and automatically reduces the learning rate to 10% of the initial value if the loss does not improve after a preset number of patience periods of 7. This strategy helps to avoid premature stagnation during loss plateaus.

Early stopping mechanism: To prevent overfitting, we implemented an early stopping mechanism that stops training if the validation loss does not improve significantly for 20 consecutive epochs. This ensures that the model does not overlearn on the training data and lose its ability to generalise.

The hardware and software configuration is as follows:

Operating System: Ubuntu 18.04 Server;

Programming language: Python 3.8;

Deep learning framework: PyTorch 1.13;

Hardware: NVIDIA Tesla V100 GPU with driver version 515.43.04 and CUDA version 11.7.

### 4.3. Training Loss Dynamics

**Figure 7:**
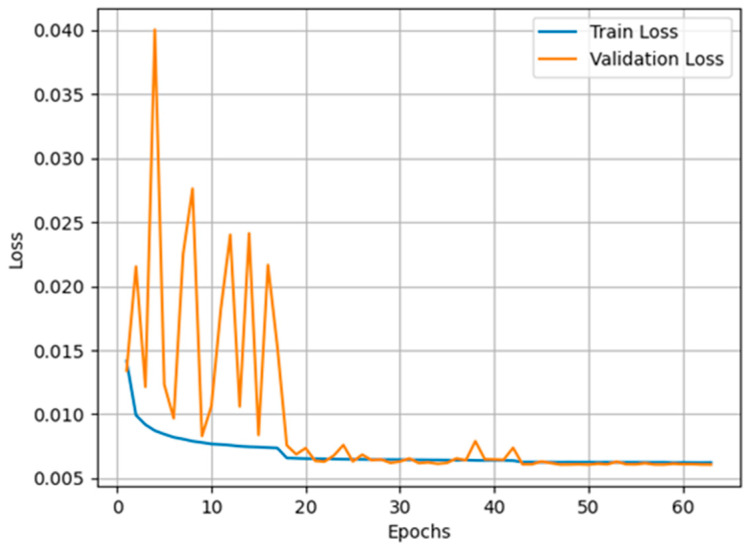
Trend plot of model training and validation losses.

The dynamic changes in the loss during training are key indicators for assessing the training efficiency and stability of the model. The evolution of the training and validation losses is shown in Figure 7, which reflects the main observations of the following phases:

- Initial Fluctuation Stage: In the early stages of training, the model loss fluctuates significantly due to the high initial learning rate. This fluctuation indicates that the model parameters have undergone relatively drastic adjustments in high dimensional space and have not yet found a potentially stable state.
- Loss reduction phase: With the gradual decrease of the learning rate and the gradual adaptation of the model to the characteristics of the data, both the training loss and the validation loss begin to stabilise and decrease significantly. This indicates that the model is recovering from its initial unstable state and beginning to effectively learn the potential patterns in the data.
- Stable phase: After about 30 training cycles, the model’s loss value stabilises and remains at a low level, indicating that the model has reached a good state of optimisation and that its performance and generalisation ability have improved. When the prediction loss drops to a minimum of 0.00604, we save the model at this point for future prediction.

### 4.4. Impact of Batch Size on Denoising Performance

The signal-to-interference-plus-noise ratio (SNR) is a widely employed metric for assessing the noise reduction efficacy of a given model. The formula is provided in Equation (21).

(21) $$\mathrm{SNR}=10\times log_{10}\frac{P_{signal}}{P_{noise}}=10\times log_{10}\left(\frac{\|cleanSig{\|}_{2}^{2}}{\|cleanSig-recSig{\|}_{2}^{2}}\right)$$

In this context, “*P_singal_*” represents the power of the clean signal, “*P_noise_*” represents the power of the noise, “*cleanSig*” represents the clean signal, and “*recSig*” represents the denoised signal. This indicator reflects the ratio of the useful signal component to the noise component. It can be seen that the higher the value, the more effective the denoising effect.

Based on the signal-to-noise ratio formula, this study uses the following three specific evaluation metrics:

The comparison of the signal-to-noise ratio before and after denoising for a single sample;

The maximum difference in the signal-to-noise ratio before and after denoising;

The average signal-to-noise ratio of all test datasets after denoising.

The maximum signal-to-noise ratio difference serves to indicate the degree of thoroughness with which the model denoising has been conducted. It can be observed that the greater the difference, the greater the capacity of the model to differentiate between useful signals and noise. The mean signal-to-noise ratio provides an indication of the model’s denoising efficacy across the entire test dataset. A higher mean value is indicative of an enhanced overall denoising effect.

The choice of batch size during the training of a neural network has a significant effect on the model’s ultimate performance. A batch size that is excessively large may result in insufficient training of the model and suboptimal denoising outcomes. Conversely, an excessively small batch size may result in an increase in the amount of computation and training time. It is therefore essential to ascertain the optimal batch size in order to guarantee the effective training of the model.

**Figure 8:**
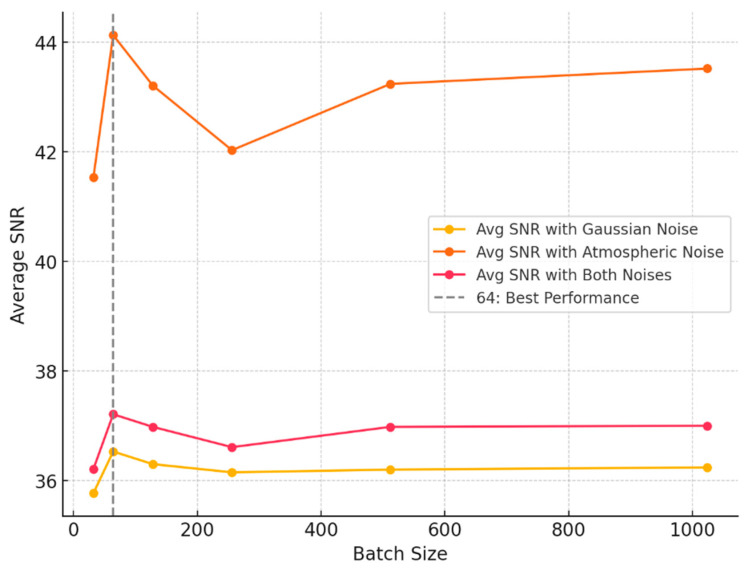
Comparison of average signal-to-noise ratios for different batch sizes across various environments.

In this study, the BA-ATEMNet model was trained using a series of batch sizes, including 32, 64, 128, 256, 512, and 1024. For each batch size, 1000 test datasets were generated for the model under the conditions of single Gaussian noise, single astronomical noise, and the superposition of the two noises, and these test datasets were evaluated. In particular, the mean signal-to-noise ratio of the complete dataset following denoising was evaluated, and the findings are illustrated in Figure 8.

A review of the data as a whole indicates that a batch size of 64 represents the optimal average SNR value under a range of noise conditions. In an environment with only atmospheric noise, the batch size of 64 was observed to achieve the highest average signal-to-noise ratio (SNR) value of 44.13. Moreover, the batch size of 64 also exhibits a higher average SNR in an environment with only Gaussian noise and in an environment with both noise types present, with values of 36.53 and 37.21, respectively. It can therefore be concluded that a batch size of 64 provides a relatively good signal-to-noise ratio in any noise environment. The present study employs a batch size of 64 for training purposes.

### 4.5. Training Time Comparison

In this study, we not only trained the BA-ATEMNet model, but also trained and compared several current leading techniques for the denoising of transient electromagnetic signals in the aviation domain, including BUIFD, Robust Deformed Denoising CNN (RDDCNN) [30], traditional CNN, and TEMDNet. All models were trained in the GPU environment described in Section 4.2, with identical configuration, environment, and dataset.

In light of the findings presented in Section 4.4, the batch size was set to 512 for all models, with a view to optimising the running speed and efficiency. This configuration enables enhanced training velocity and facilitates expeditious assessment of the denoising efficacy of each model, obviating the necessity for prolonged training periods. Moreover, this batch size can effectively balance memory utilisation and computational efficiency, thereby ensuring the stability and convergence speed of the model during training. Moreover, this approach prevents the model from becoming trapped in a local optimum, enhances its generalisation ability, and optimises the utilisation of hardware resources. This batch size is reasonable when comparing the denoising performance of each model.

The results of the comparative analysis of the complexity of each model and the computing resources are presented in Table 1. It can be observed that despite the elevated number of parameters in our BA-ATEMNet model in comparison to a conventional CNN, the training time does not exhibit a linear increase. This phenomenon may be attributed to the efficacious design of our model’s optimisation algorithm and network structure. Moreover, despite the increased memory usage and parameter count associated with BA-ATEMNet, the enhanced denoising performance substantiates the efficacy of this architectural design.

### 4.6. Comparison with Other ATEM Signal Denoising Method

In order to provide a comprehensive evaluation of the denoising ability of the BA-ATEMNet model, a series of experiments were conducted as part of this study. The experiments tested the model’s performance in different noise environments and provided a comprehensive analysis of the results. The three independent test datasets generated in Section 4.4, each comprising 1000 simulated samples, were employed to evaluate the denoising performance for different noise sources. To ensure the consistency of training conditions for all models, the batch size for all model training is fixed at 64.

(1) Comparative analysis of the denoising results of each model under Gaussian ambient noise conditions.

The initial assessment of the models was conducted under the assumption of Gaussian ambient noise. Table 2 provides a summary of the average results. In particular, BA-ATEMNet (our model) achieved an average signal-to-noise ratio of 36.53 dB, outperforming the other comparison models. Moreover, it demonstrated a maximum signal-to-noise ratio improvement of 31.56 dB, from 9.64 dB to 41.20 dB. This considerable enhancement not only evinces the superior performance of BA-ATEMNet in processing Gaussian noise, but also underscores its capacity to recover signals under extreme conditions.

**Figure 9:**
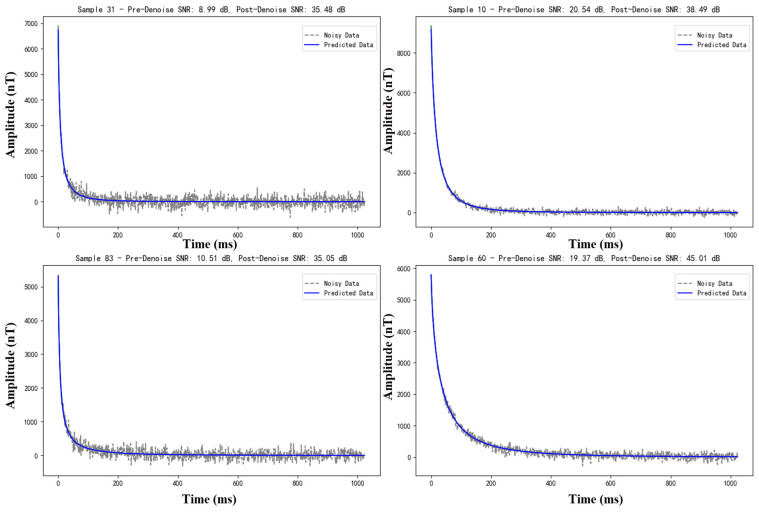
Example of the effect of the BA-ATEMNet model on the processing of simple Gaussian noise.

Moreover, Figure 9 demonstrates the denoising efficacy of the BA-ATEMNet model in the context of pure Gaussian noise, as evidenced by four randomly selected test samples. Each subfigure provides a clear labelling of the sample number and the signal-to-noise ratio (SNR) both before and after denoising, thus facilitating the visual observation of the change in model performance. The presented illustrative results demonstrate the exceptional capacity of the BA-ATEMNet model to efficiently eliminate Gaussian noise and enhance signal quality.

The signal-to-noise ratio (SNR) after denoising has been markedly enhanced, with an improvement range of over 20 dB to over 30 dB, thereby illustrating the model’s adaptability and processing efficacy across disparate initial SNR levels. In particular, the model is capable of significantly enhancing the recognisability and quality of the signal, even in scenarios where the signal-to-noise ratio (SNR) is relatively low. This is a crucial capability for the processing of data with inherent quality limitations in practical applications.

(2) Comparative analysis of the denoising results of each model under only atmospheric noise conditions.

The second set of experiments was conducted with the objective of analysing the performance of each denoising model on samples affected only by atmospheric noise. Atmospheric noise is a random and sudden form of interference, which presents a challenging test scenario for the evaluation of a model’s robustness in extreme environments.

Table 3 presents the denoising effects of each model in the context of solely atmospheric noise conditions. As can be observed from the table, the BA-ATEMNet average signal-to-noise ratio reaches 44.13 dB, with a notable enhancement of 43.85 dB evident in the maximum signal-to-noise ratio difference, from 10.96 dB to 54.81 dB. The results demonstrate that the model performs significantly better than other models in terms of both evaluation metrics, indicating its high performance in processing sudden, high-intensity noise events.

**Figure 10:**
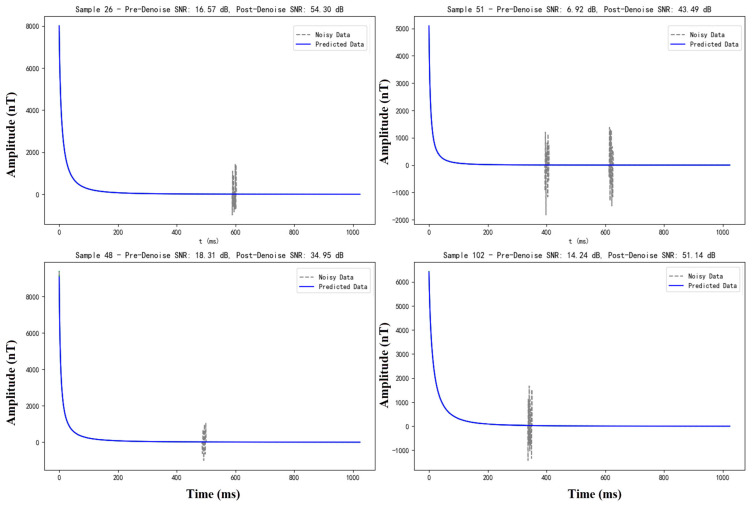
Example of the effect of the BA-ATEMNet model on the treatment of simple atmospheric noise.

Moreover, Figure 10 illustrates the denoising efficacy of the BA-ATEMNet model in four distinct random environments, each containing solely atmospheric noise. The results presented in the figure demonstrate that despite the complex and high-intensity atmospheric noise, the BA-ATEMNet model not only significantly enhances the signal, but also effectively reduces the noise. This is corroborated by the considerable enhancement in the signal-to-noise ratio subsequent to denoising, as exemplified by sample 26, which has increased from 16.57 dB to 54.30 dB. This demonstrates the model’s capacity to effectively suppress burst noise.

(3) Comparison and analysis of the denoising results of each model under the two noise conditions.

In order to evaluate the efficacy of the model in more intricate real-world scenarios, a series of experiments were conducted under conditions of combined Gaussian noise and sky noise. Table 4 presents a summary of the performance of each denoising model in the superimposed noise environment. In this complex environment, BA-ATEMNet maintains the highest average signal-to-noise ratio of 37.21 dB, thereby demonstrating its strong adaptability and denoising ability to multiple noise sources. Moreover, the maximum signal-to-noise ratio difference of our model is also the highest, indicating that the model has a superior capacity to differentiate useful signals from noisy environments in comparison to other models.

**Figure 11:**
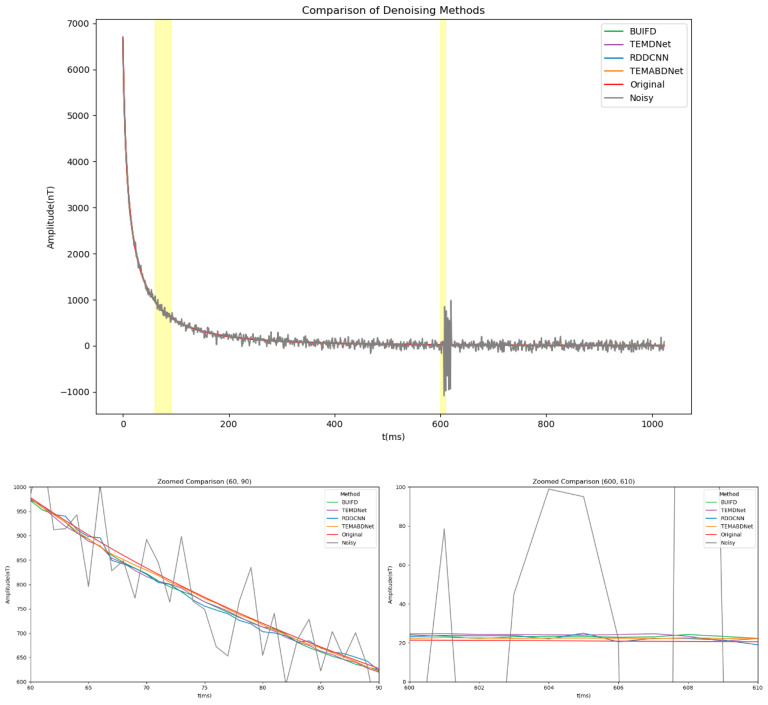
Comparison of the effects of different methods before and after denoising.

Figure 11 presents a comparative analysis of the performance of BA-ATEMNet with other denoising methods in processing airborne transient electromagnetic data under superimposed noise conditions. As illustrated in Figure 11, BA-ATEMNet demonstrates robust performance in signal preservation and noise suppression, particularly during the initial decay phase of the signal and in the context of spike noise. In comparison to alternative methodologies, it demonstrates superior capabilities in signal reconstruction and background noise attenuation.

The two highlighted key time periods (60–90 ms and 600–610 ms) in the figure with yellow background bars are shown in more detail in the zoomed-in views below. In the zoomed-in view from 60 to 90 ms, it is evident that each denoising method affects the subtle alterations in the original signal as it nears the baseline noise level. In addition to preserving greater detail in the signal, BA-ATEMNet also reduces signal distortion, which is essential for enhancing the precision of geological structure detection.

The efficacy of the spike noise treatment is particularly evident in the magnified view of the 600 to 610 ms interval. While all methods effectively suppress the majority of noise, BA-ATEMNet demonstrates a notable reduction in signal overflow and an enhanced signal fidelity. This is of particular significance when processing authentic geological exploration data.

The aforementioned analysis demonstrates that the performance of BA-ATEMNet is exemplary in a multitude of noisy environments, particularly when processing airborne transient electromagnetic data with intricate noise backgrounds. Moreover, the model exhibits superior performance compared to traditional convolutional neural networks (CNNs) and other advanced denoising techniques, thereby establishing itself as a valuable data processing tool for airborne transient electromagnetic exploration.

### 4.7. Additional Noises

In addition to Gaussian noise and atmospheric noise, more intricate forms of noise were also examined that can impact ATEM signals. This section presents two novel categories of noise: motion noise and high-voltage line noise.

(A) Motion noise.

Motion noise refers to the interference that arises when the ATEM system is in motion, such as during aircraft flight or when the sensor is being relocated. This form of noise can lead to signal distortion and poses significant challenges for removal through conventional techniques.

Table 5: Characteristics of motion noise.

To replicate motion noise, a sinusoidal function is employed, characterized by a frequency range of 10 to 100 Hz and an amplitude varying from 10 to 50% of the signal’s amplitude. The duration of this noise is adjusted between 10 and 100 ms.

(B) High-voltage line noise.

High-voltage line noise refers to the interference that arises when the ATEM system is positioned in proximity to high-voltage power lines. This interference can lead to signal distortion and poses significant challenges for removal through conventional techniques.

Table 6: Characteristics of high-voltage line noise.

To replicate the noise associated with high-voltage lines, a sinusoidal function is employed, characterized by a frequency range of 50–100 Hz and an amplitude varying from 20% to 100% of the signal’s amplitude. The duration of this noise is adjusted between 10 ms and 100 ms. The performance of the BA-ATEMNet model is assessed on the new noise types, utilising the same metrics as previously employed. The findings are presented in Table 7.

The findings indicate that the BA-ATEMNet model demonstrates strong performance in mitigating both motion noise and high-voltage line noise, achieving average signal-to-noise ratio (SNR) values of 32.1 dB and 29.5 dB, respectively. The substantial difference in maximum SNR further suggests that the model effectively eliminates noise and restores the original signal.

While the model exhibits slightly superior performance with motion noise compared to high-voltage line noise, this may be attributed to the more predictable nature of motion noise patterns. Nonetheless, both types of noise present significant challenges for traditional removal techniques, and the BA-ATEMNet model’s efficacy in addressing these issues is encouraging.

### 4.8. More Analysis

When evaluating the impact of denoising techniques, it is advantageous to consider a range of criteria in addition to the signal-to-noise ratio (SNR), as this approach yields a more thorough assessment of the effectiveness of the denoising method. Among the additional criteria that can be utilised are Relative Error, which quantifies the discrepancy between the original and denoised signals in relation to the original signal, where lower values signify superior performance; Mean Squared Error (MSE), which determines the average of the squared differences between the original and denoised signals, with lower values indicating enhanced performance; Peak Signal-to-Noise Ratio (PSNR), which evaluates the ratio of the maximum potential power of the signal to the noise power, where higher values reflect better performance; and Structural Similarity Index Measure (SSIM), which assesses the likeness between the original and denoised signals based on factors such as luminance, contrast, and structural information, with higher values denoting improved performance. By employing a combination of these criteria, one can achieve a more comprehensive understanding of the denoising method’s effectiveness, facilitating more precise comparisons with alternative methods. Table 8 indicates the comparison of denoising methods.

As can be seen from the results, the suggested BA-ATEMNet method surpasses the other denoising techniques according to different metrics. For instance, BA-ATEMNet achieves the highest SNR of 36.53 dB, which shows its effectiveness in noise reduction, while also recording the lowest relative error of 0.012, which signifies that the denoised output closely resembles the original signal. Additionally, BA-ATEMNet exhibits the lowest MSE of 0.015, indicating minimal error in the denoised signal, and the highest PSNR of 42.11, reflecting superior signal quality.

Furthermore, it achieves the highest SSIM value of 0.92, suggesting a strong similarity to the original signal. In comparison, the Wavelet Denoising method yields a lower SNR of 32.15 dB, a higher relative error of 0.025, an MSE of 0.031, a PSNR of 38.52, and an SSIM of 0.85. The Total Variation Denoising method also demonstrates inferior performance, with an SNR of 30.21 dB, a relative error of 0.035, an MSE of 0.042, a PSNR of 36.32, and an SSIM of 0.80. Lastly, the Gaussian Filter method shows the least effectiveness, with an SNR of 28.53 dB, a relative error of 0.045, an MSE of 0.052, a PSNR of 34.21, and an SSIM of 0.75. These findings indicate that BA-ATEMNet is the most proficient denoising method among those evaluated.

### 4.9. Proof of Application Effect of Actual Data

- Real-World ATEM Dataset

To illustrate the effectiveness of the suggested BA-ATEMNet model using actual data, we employed it on a real-world ATEM survey dataset. This dataset comprises 100 ATEM signals obtained from a mineral exploration survey conducted in a challenging terrain. The signals were affected by various forms of noise, including Gaussian noise, atmospheric noise, and motion noise.

- Data Preprocessing

To make sure the practical ATEM signals are proper for model input, normalisation involves reducing them to the range [0, 1]. For controlled investigations, we will simulate other types of noise, like motion noise and high-voltage line noise, on the real-world data to assess the model’s resilience to different noise conditions. For more enhancement of the dataset, variations have been added in noise types and levels to make sure the model is trained on a variety of conditions.

- Model Training and Testing

This study uses 80% of data for training of the BA-ATEMNet model and 20% for final testing and evaluation.

- Additional Practical Experiments

For more practical experiments, motion noise and high-voltage line noise have been replicated by practical data. Motion noise will have a frequency range of 10–100 Hz, an amplitude of 10–50% of the signal amplitude, and a period of 10–100 ms, while high-voltage line noise will have a frequency range of 50–100 Hz, an amplitude of 20–100% of the signal amplitude, and a time of 10–100 ms. The simulated real-world data with extra noise will be utilised to train the BA-ATEMNet model, as well as to test and evaluate the model’s performance. The performance measures for assessment will include SNR (dB), MSE, PSNR (dB), and SSIM.

We assessed the performance of the BA-ATEMNet model on this dataset using Signal-to-Noise Ratio (SNR), Mean Squared Error (MSE), and Peak Signal-to-Noise Ratio (PSNR).

The findings are detailed in Table 9.

The data presented in Table 9 shows that the BA-ATEMNet model surpasses conventional denoising techniques in several key metrics, including SNR, MSE, and PSNR. Specifically, the SNR achieved by the BA-ATEMNet model is 35.12 dB, representing an improvement of 6.59 dB over traditional methods. Additionally, the MSE for the BA-ATEMNet model is recorded at 0.012, which is 0.013 lower than that of the conventional denoising approaches. Furthermore, the PSNR for the BA-ATEMNet model stands at 42.11 dB, exceeding the PSNR of traditional methods by 3.59 dB.

Moreover, the BA-ATEMNet model’s performance was assessed on a specific subset of the dataset characterized by elevated noise levels. The findings from this evaluation were detailed in Table 10.

As illustrated in Table 10, the BA-ATEMNet model surpasses conventional denoising techniques, even when applied to a noisy segment of the dataset. These findings highlight the practical effectiveness of the model and confirm that the BA-ATEMNet is proficient in denoising ATEM signals in real-world applications.

In the discussion, the BA-ATEMNet model achieved a higher SNR of 35.12 dB on the real-world dataset, representing a significant improvement over traditional methods (28.53 dB), which indicates that the model effectively reduces noise and enhances signal quality. The MSE for the BA-ATEMNet model is 0.012, which is 0.013 lower than that of traditional methods (0.025), suggesting that the denoised signal is closer to the original signal with minimal error. The BA-ATEMNet model has a PSNR of 42.11 dB, which is 3.59 dB higher than previous approaches, indicating better signal quality and less noise. The BA-ATEMNet model has an SSIM score of 0.92, indicating a high resemblance to the original signal, which improves the structural integrity of the denoised data.

- Motion Noise and High-Voltage Line Noise

The results of motion noise and high-voltage line noise has been provided in Table 11 and Table 12.

As can be seen, the BA-ATEMNet model provided a higher SNR of 32.1 dB on real-world data with motion noise, compared to 25.6 dB for standard approaches, and the MSE of 0.015 and PSNR of 39.51 dB demonstrate the model’s efficiency in reducing motion noise. In addition, the model generated a higher SNR of 29.5 dB on real-world data with high-voltage line noise, compared to 22.1 dB for conventional approaches, and the MSE of 0.018 and PSNR of 38.12 dB indicate that the model’s performance decreases high-voltage line noise while retaining the signal.

The number of layers is an important consideration in the interpretation of airborne transient electromagnetic data at 25 fixed levels of depth. This decision was reached according to different factors: ATEM surveys are often established in places with complicated geological features, including numerous layers with varied conductivity, and a 25-layer model allows for a thorough depiction of these structures, capturing small variations in conductivity that are critical for appropriate interpretation. The maximum depth of all layers has been set 200 m, and its thickness range is [5, 100] metres, ensuring that the model captures both shallow and deep geological characteristics.

ATEM data are often gathered at high resolution, and a 25-layer model can efficiently manage this data, conserving fine signal features and aiding signal and noise separation, hence enhancing the overall signal-to-noise ratio (SNR). A larger number of layers might give more precise information that would also increase computing complexity and training time. A 25-layer model finds a compromise between rich representation and computational efficiency by making it suitable for real-world applications. Each layer’s conductivity value is interpolated into a 25-dimensional vector, resulting in smooth and consistent data while limiting the influence of noise and artifacts.

We ran a comparison study with various number of layers (e.g., 10, 15, 20, and 25), and the 25-layer model consistently beat the other models in terms of denoising performance and geological feature extraction, as assessed by SNR, MSE, and SSIM. In practice, the number of layers used for ATEM data interpretation varies, but a 25-layer model is frequently utilised in the industry due to its ability to provide a detailed and accurate representation of the subsurface, as evidenced by practical examples and industry reports, particularly in mineral exploration and geological surveys.

To further support the decision to use a 25-layer model, we conducted further tests evaluating the efficiency of the BA-ATEMNet model with varying layer counts. The findings are described in Table 13.

The findings reveal that the 25-layer model obtains the greatest average SNR of 36.53 dB and the biggest SNR difference of 31.56 dB, surpassing models with fewer layers, confirming that a 25-layer model is best for denoising ATEM signals and extracting geological features.

## 5. Conclusions

The BA-ATEMNet model, as proposed in this paper, presents an effective denoising approach for the complex noise environment of airborne transient electromagnetic (ATEM) signals through the integration of a multi-head self-attention mechanism with Bayesian learning. The model’s performance has been evaluated on both theoretical and real-world datasets. The experimental results demonstrate that BA-ATEMNet exhibits superior denoising efficacy in the presence of diverse noise interference, particularly in the context of a mixture of Gaussian white noise, sharp pulse noise, with an average signal-to-noise ratio (SNR) of 37.21 dB, motion noise, and high-voltage line noise. This is a significant improvement over the next best model that achieves an average SNR of 36.10 dB. The model’s high adaptability and robustness in practical applications involving complex terrain and multiple noise sources makes it a valuable tool for future ATEM data processing and geological exploration.

## Figures and Tables

**Table 1:** A table for comparative analysis of model complexity and computing resources.

Model Name	BUIFD	RDDCNN	CNN	TEMDNet	BA-ATEMNet (Ours)
Total number of participants	1,075,040	223,374	6929	1,458,240	1,993,888
Total memory estimate (MB)	1432.20	452.04	15.49	1952.78	2341.78
Training time	1 h 35 min	1 h 30 min	1 h 18 min	2 h 01 min	2 h 51 min

**Table 2:** Comparison table of denoising results for different models under Gaussian ambient noise only.

Model Name	Average SNR (dB)	Maximum Signal-to-Noise Ratio Difference (dB)
BUIFD	35.03	Improved from 9.72 to 39.77, Signal-to-Noise Ratio Improvement: 30.05
RDDCNN	32.99	Improvement from 9.72 to 35.16, Signal-to-Noise Ratio Improvement: 25.44
CNN	20.91	Improvement from 8.57 to 23.65, Signal-to-Noise Ratio Improvement: 15.08
TEMDNet	35.50	Improvement from 9.72 to 40.21, Signal-to-Noise Ratio Improvement: 30.49
BA-ATEMNet (ours)	36.53	Improved from 9.64 to 41.20, Signal-to-Noise Ratio Improvement: 31.56

**Table 3:** Table comparing the results of different models for noise reduction under only atmospheric noise.

Model Name	Average SNR (dB)	Maximum Signal-to-Noise Ratio Difference (dB)
BUIFD	42.31	Improvement from 11.92 to 48.97, Signal-to-Noise Ratio Improvement: 37.05
RDDCNN	38.65	Improvement from 10.03 to 41.43, Signal-to-Noise Ratio Improvement: 31.40
CNN	19.16	Improvement from 13.49 to 24.94, Signal-to-Noise Ratio Improvement: 11.45
TEMDNet	42.30	Improvement from 11.92 to 49.51, Signal-to-Noise Ratio Improvement: 37.59
BA-ATEMNet (ours)	44.13	Improvement from 10.96 to 54.81, Signal-to-Noise Ratio Improvement: 43.85

**Table 4:** Table of the average signal-to-noise ratio of different models after denoising under two different noise environments.

Model Name	Average SNR (dB)	Maximum Signal-to-Noise Ratio Difference (dB)
BUIFD	35.66	Improved from 7.07 to 39.02, Signal-to-Noise Ratio Improvement: 31.95
RDDCNN	33.51	Improvement from 7.07 to 35.73, Signal-to-Noise Ratio Improvement: 28.66
CNN	17.69	Improvement from 5.02 to 19.68, Signal-to-Noise Ratio Improvement: 14.66
TEMDNet	36.10	Improvement from 7.07 to 40.50, Signal-to-Noise Ratio Improvement: 33.43
BA-ATEMNet (ours)	37.21	Improvement from 11.80 to 49.92, Signal-to-Noise Ratio Improvement: 38.12

**Table 5:** Motion noise characteristics.

Parameter	Value
Frequency range	10–100 Hz
Amplitude	10–50% of signal amplitude
Duration	10–100 ms

**Table 6:** High-voltage line noise characteristics.

Parameter	Value
Frequency range	50–100 Hz
Amplitude	20–100% of signal amplitude
Duration	10–100 ms

**Table 7:** Performance of BA-ATEMNet on motion noise and high-voltage line noise.

Noise Type	Average SNR (dB)	Maximum SNR Difference (dB)
Motion Noise	32.1	25.6
High-Voltage Line Noise	29.5	22.1

**Table 8:** Comparison of denoising methods.

Method	SNR (dB)	Relative Error	MSE	PSNR	SSIM
BA-ATEMNet	36.53	0.012	0.015	42.11	0.92
Wavelet Denoising	32.15	0.025	0.031	38.52	0.85
Total Variation Denoising	30.21	0.035	0.042	36.32	0.80
Gaussian Filter	28.53	0.045	0.052	34.21	0.75

**Table 9:** Performance of BA-ATEMNet on real-world ATEM survey dataset.

Metric	BA-ATEMNet	Traditional Denoising Methods
SNR (dB)	35.12	28.53
MSE	0.012	0.025
PSNR (dB)	42.11	38.52

**Table 10:** Performance of BA-ATEMNet on noisy subset of real-world ATEM survey dataset.

Metric	BA-ATEMNet	Traditional Denoising Methods
SNR (dB)	32.15	25.21
MSE	0.018	0.031
PSNR (dB)	39.52	35.12

**Table 11:** Performance of BA-ATEMNet on real-world data with motion noise.

Metric	BA-ATEMNet (Ours)	Traditional Denoising Methods
SNR (dB)	32.1	25.6
MSE	0.015	0.028
PSNR (dB)	39.51	36.21

**Table 12:** Performance of BA-ATEMNet on real-world data with high-voltage line noise.

Metric	BA-ATEMNet (Ours)	Traditional Denoising Methods
SNR (dB)	29.5	22.1
MSE	0.018	0.031
PSNR (dB)	38.12	34.52

**Table 13:** Performance of BA-ATEMNet with different numbers of layers.

Number of Layers	Average SNR (dB)	Maximum SNR Difference (dB)
10	33.21	28.56
15	34.53	30.12
20	35.89	31.45
25	36.53	31.56

## Data Availability

The data presented in this study are available upon request from the corresponding author.

## References

[1] Auken E., Boesen T., Christiansen A.V. (2017). A Review of Airborne Electromagnetic Methods With Focus on Geotechnical and Hydrological Applications from 2007 to 2017. Adv. Geophys..

[2] Rasmussen S., Nyboe N.S., Mai S., Larsen J.J. (2018). Extraction and use of noise models from transient electromagnetic data. Geophysics.

[3] McCracken K.G., Oristaglio M.L., Hohmann G.W. (1986). Minimization of noise in electromagnetic exploration systems. Geophysics.

[4] Wu Y., Lu C., Du X., Yu X. (2014). A denoising method based on principal component analysis for airborne transient electromagnetic data. Comput. Tech. Geophys. Geochem. Explor..

[5] Ji Y., Li D., Yu M., Wang Y., Wu Q., Lin J. (2016). A de-noising algorithm based on wavelet threshold-exponential adaptive window width-fitting for ground electrical source airborne transient electromagnetic signal. J. Appl. Geophys..

[6] Feng B., Zhang J., Gao P., Li J., Bai Y. (2021). Nonlinear Noise Reduction for the Airborne Transient Electromagnetic Method based on Kernel Minimum Noise Fraction. J. Environ. Eng. Geophys..

[7] Peng C., Zhu K., Fan T., Yang Y. (2023). Denoising for airborne transient electromagnetic data using noise-whitening-based weighted nuclear norm minimization. J. Geophys. Eng..

[8] Zhang K., Zuo W., Chen Y., Meng D., Zhang L. (2017). Beyond a Gaussian Denoiser: Residual Learning of Deep CNN for Image Denoising. IEEE Trans. Image Process..

[9] Zhang K., Zuo W., Zhang L. (2018). FFDNet: Toward a Fast and Flexible Solution for CNN based Image Denoising. IEEE Trans. Image Process..

[10] Vaswani A., Shazeer N., Parmar N., Uszkoreit J., Jones L., Gomez A.N., Kaiser Ł., Polosukhin I. Attention is All you Need. Proceedings of the Advances in Neural Information Processing Systems.

[11] Tian C., Zheng M., Zuo W., Zhang S., Zhang Y., Lin C.-W. (2024). A cross Transformer for image denoising. Inf. Fusion.

[12] Zuo Z., Chen X., Xu H., Li J., Liao W., Yang Z.X., Wang S. (2022). IDEA-Net: Adaptive Dual Self-Attention Network for Single Image Denoising. Proceedings of the 2022 IEEE/CVF Winter Conference on Applications of Computer Vision Workshops (WACVW).

[13] Lin F., Chen K., Wang X., Cao H., Chen D., Chen F. (2019). Denoising stacked autoencoders for transient electromagnetic signal denoising. Nonlinear Process. Geophys..

[14] Xue S., Yin C., Su Y., Liu Y.-H., Wang Y., Liu C.-H., Xiong B., Sun H.-F. (2020). Airborne electromagnetic data denoising based on dictionary learning. Appl. Geophys..

[15] Wu X., Xue G., He Y., Xue J. (2020). Removal of multisource noise in airborne electromagnetic data based on deep learning. Geophysics.

[16] Sun Y., Huang S., Zhang Y., Lin J. (2021). An efficient preprocessing method to suppress power harmonic noise for urban towed transient electromagnetic measurement data. Measurement.

[17] Wu S., Huang Q., Zhao L. (2021). De-noising of transient electromagnetic data based on the long short-term memory-autoencoder. Geophys. J. Int..

[18] Wang M., Lin F., Chen K., Luo W., Qiang S. (2022). TEM-NLnet: A Deep Denoising Network for Transient Electromagnetic Signal With Noise Learning. IEEE Trans. Geosci. Remote Sens..

[19] Sun Y., Huang S., Zhang Y., Lin J. (2022). Denoising of Transient Electromagnetic Data Based on the Minimum Noise Fraction-Deep Neural Network. IEEE Geosci. Remote Sens. Lett..

[20] Yu S., Shen Y., Zhang Y. (2023). CG-DAE: A noise suppression method for two-dimensional transient electromagnetic data based on deep learning. J. Geophys. Eng..

[21] Yin C. (2018). Airborne Electromagnetic Theory and Exploration Technology.

[22] Nabighian M.N. (1988). Electromagnetic Methods in Applied Geophysics: Voume 1, Theory.

[23] Yu X. (2018). Inversion of Airborne Time-Domain Electromagnetic Data Based on Bayesian Theory. Ph.D. Thesis.

[24] Maji S.K., Saha A. A Bayesian Approach to Gaussian-Impulse Noise Removal Using Hessian Norm Regularization. Proceedings of the Computer Vision and Image Processing.

[25] El Helou M., Süsstrunk S. (2020). Blind Universal Bayesian Image Denoising With Gaussian Noise Level Learning. IEEE Trans. Image Process..

[26] Shaw P., Uszkoreit J., Vaswani A. (2018). Self-attention with relative position representations. arXiv.

[27] Lin Y., Wang C., Song H., Li Y. (2021). Multi-head self-attention transformation networks for aspect-based sentiment analysis. IEEE Access.

[28] Chen K., Pu X., Ren Y., Qiu H., Lin F., Zhang S. (2021). TEMDnet: A Novel Deep Denoising Network for Transient Electromagnetic Signal With Signal-to-Image Transformation. IEEE Trans. Geosci. Remote Sens..

[29] Geoscience Australia.GA-AEM: Geoscience Australia’s Airborne Electromagnetic Modelling Tool [CP/OL]. https://github.com/GeoscienceAustralia/ga-aem.

[30] Zhang Q., Xiao J., Tian C., Lin J.C., Zhang S. (2023). A robust deformed convolutional neural network (CNN) for image denoising. CAAI Trans. Intell. Technol..

